# Solvent-Antisolvent Ambient Processed Large Grain Size Perovskite Thin Films for High-Performance Solar Cells

**DOI:** 10.1038/s41598-018-31184-0

**Published:** 2018-08-27

**Authors:** Dawit Gedamu, Ivy M. Asuo, Daniele Benetti, Matteo Basti, Ibrahima Ka, Sylvain G. Cloutier, Federico Rosei, Riad Nechache

**Affiliations:** 1 École de technologie supérieure (ÉTS), Department of Electrical Engineering, 1100 rue Notre-Dame Ouest, Montréal, (QC) H3C 1K3 Canada; 2 INRS-EMT Centre for Energy, Materials and Telecommunication, 1650 Boul. Lionel Boulet, Varennes, (QC) J3X 1S2 Canada; 3 Institute of Fundamental and Frontier Science, University of Electronic Science and Technology of China, Chengdu, 610054 PR China

## Abstract

In recent years, hybrid organic-inorganic halide perovskites have been widely studied for the low-cost fabrication of a wide range of optoelectronic devices, including impressive perovskite-based solar cells. Amongst the key factors influencing the performance of these devices, recent efforts have focused on tailoring the granularity and microstructure of the perovskite films. Albeit, a cost-effective technique allowing to carefully control their microstructure in ambient environmental conditions has not been realized. We report on a solvent-antisolvent ambient processed CH_3_NH_3_PbI_3−*x*_Cl_*x*_ based thin films using a simple and robust solvent engineering technique to achieve large grains (>5 µm) having excellent crystalline quality and surface coverage with very low pinhole density. Using optimized treatment (75% chlorobenzene and 25% ethanol), we achieve highly-compact perovskite films with 99.97% surface coverage to produce solar cells with power conversion efficiencies (PCEs) up-to 14.0%. In these planar solar cells, we find that the density and size of the pinholes are the dominant factors that affect their overall performances. This work provides a promising solvent treatment technique in ambient conditions and paves the way for further optimization of large area thin films and high performance perovskite solar cells.

## Introduction

Hybrid organic-inorganic perovskite materials of the form ABX_3_ (where A = CH_3_NH_3_, FA, or FA_y_(CH_3_NH_3_)_1−y_ B = Pb, Sn, X = I, Cl) have recently emerged as promising material systems because of their low-cost synthesis and wide range of optoelectronic applications including light emitting diodes (LED)^1–4^, photodetectors^5^ and solar cells^6–8^. Their excellent properties such as high photon absorption, tunable bandgap and versatile material properties and fabrication processes^9^ allow them to compete with the established semiconductor-based solar cell technologies. Currently, physical deposition methods including pulsed laser deposition^10–12^ and chemical routes are mostly employed in the deposition of light absorber materials on transparent conductive substrates such as fluorine-doped tin oxide (FTO) or indium-doped tin oxide (ITO). Using chemical routes, perovskite films can be deposited using standard processes with a single or two-step spin-coating process on FTO or ITO coated glass substrates. Significant progress has also been achieved without using scaffolds, thereby minimizing materials and processing costs^13^. However, operational device stability and the substitution of lead (Pb)^14,15^ remain the two major challenges associated with the success of commercial-grade perovskite-based solar cell devices^16^.

In the last few years, perovskite solar cells (PSCs) have shown very rapid increase in power conversion efficiency (PCE) since the pioneering work of Miyaska *et al*.^6^. PCEs exceeding 20% have been achieved for three dimensional perovskite-based solar cells^17–20^. A record efficiency of 22.1% and excellent stability have been reported using mesoporous scaffolds deposited on charge extractors so that the perovskite would infiltrate the pores^21^. To enhance efficiency and improve device stability, materials such as TiO_2_, SnO_2_, phenyl-C61-butyric acid methyl ester (PCBM) as electron extractors and polymers 2,2′,7,7′-tetrakis (*N*,*N*-di-*p*-methoxyphenyl-amine) 9,9′-spirobifluorene (Spiro-MeOTAD) poly(3,4-ethylenedioxythiophene)/poly(4-styrenesulfonate) (PEDOT:PSS) as hole extractors have been implemented using various deposition techniques^9,15,20,22,23^. The stability of PSCs exposed to 55–70% humidity range has been shown to improve by introducing chemical additives such as a thiocyanate (Pb(SCN)_2_)^16^ or phosphoric acid ω-ammonium chlorides^24^ in the perovskite precursor solutions.

The film microstructure including grain size^25,26^, grain boundaries^27^, density of pinholes, crystalline quality and orientation of the film also significantly affect the performance of the solar cells. In particular, fabrication in ambient conditions while maintaining full control over the microstructure of the film is a major technical challenge^28,29^. To this end, numerous recent efforts focused on tailoring perovskite grain size and microstructure with specific morphologies. Small crystal grains were found to suppress exciton formation^28^, and methods such as sulfonate carbon nanotubes filled grain boundaries^30^, or Pb(SCN)_2_^31^ additives result in homogenous films with larger grain sizes and subsequently increase the PCEs. Using various antisolvents (toluene, 2-propanol, chlorobenzene)^7,21,32,33^ and mixed antisolvent (chlorobenzene:2-propanol mix)^34^, perovskite microstructure can also be tailored. However, a cost-effective technique to control the microstructure under ambient conditions has not been realized yet^35^. A simple and robust processing technique to tailor the crystallization process and reproducibly fabricate highly-crystalline pinhole-free films with large grain sizes is a necessary step towards efficient PSCs that can be scaled-up for commercialization.

In this report, we address the key challenge of controlling the microstructure of the perovskite films processed in ambient conditions by using a cost-effective solvent treatment approach to synthesize highly-oriented crystallites with statistically-controlled grain sizes and low pinhole densities. This approach yields high quality films with 5 µm grains and minimized grain boundaries. Yet, all the active layers are deposited in ambient environment (RH ~ 40%) using a precursor solution of 1:1:4 molar ratios of PbI_2_, PbCl_2_ and Methyl Ammonium Iodide (MAI). Combinations of ethanol (EtOH) and chlorobenzene (CB) in various volumetric proportions are used to treat the film at the third spin-coating (solvent dripping) step in the perovskite coating process. The solar cells fabricated using different solvent treatments indicate that the grain size and microstructure can be controllably altered, with great impact on the performance of the solar cells. Indeed, we demonstrate the effect of pinhole densities and sizes on the shunt resistance^36^, which in-turn affects the fill factor (FF) and the PCE of the devices. Using an optimized solvent treatment promoting films with larger grains and lower pinhole densities, PCEs up-to 14% are achieved for planar solar cells processed in ambient conditions. This novel solvent engineering approach yields significantly-improved crystal grain sizes and low pinhole densities, yet leaving room for further optimization. For simple planar PSC architectures, this work represents an important step towards the realization of high-quality perovskite film for low-cost and high-performance PSC devices.

## Result and Discussion

**Figure 1:**
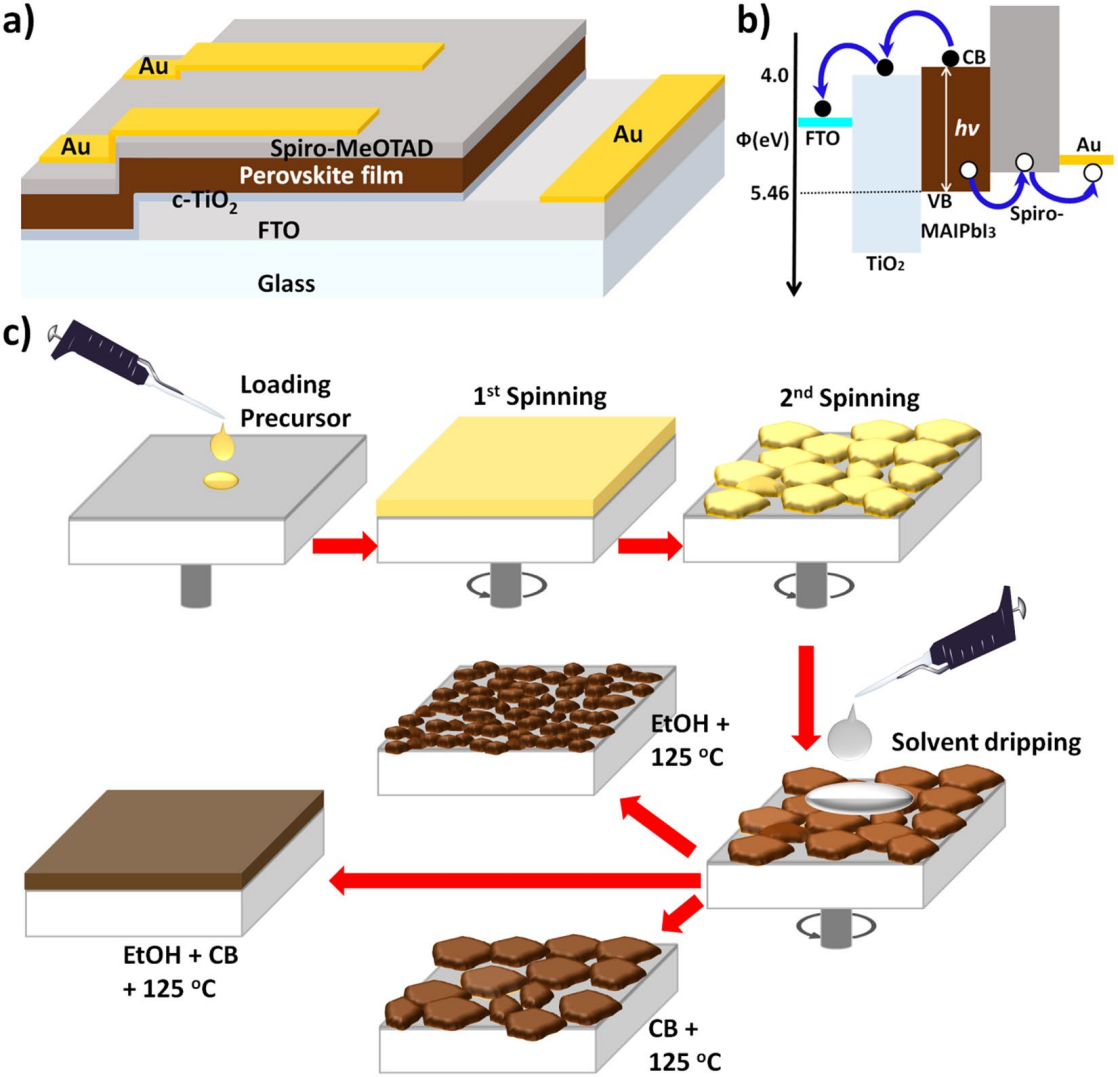
Schematic illustration of (**a**) final device architecture (**b**) energy band alignment of charge extractors to energy levels of halide perovskite where the conduction band minimum is aligned to electron injection into c-TiO_2_ and the valence band maximum is aligned to injection of holes into Spiro-MeOTAD. The processing steps in the device fabrication are depicted in the schematics (**c**) where the process begins with the perovskite precursor loading on the c-TiO_2_ coated FTO substrate followed by two spinning processes: reducing film thickness and solvent dripping (treatment) steps followed by thermal annealing for solvent removal and crystallization.

Sequential spin-coating process from solution is the most commonly-used technique for deposition of multi-layered thin films in the fabrication of heterojunction PSCs. In this work, we employ a simple one-step CH_3_NH_3_PbI_3−*x*_Cl_*x*_ perovskite solution prepared by dissolving PbI_2_, PbCl_2_ and MAI components in N,N-dimethylformamide (DMF) solvent. The CH_3_NH_3_PbI_3−*x*_Cl_*x*_ precursor solution is then spin-coated on FTO substrate atop a pre-deposited compact titanium dioxide (c-TiO_2_) layer acting as an electron-transporting layer (ETL). Following perovskite crystallization by thermal annealing, a spiro-MeOTAD layer is spin-coated in ambient condition on atop of the perovskite film to serve as a hole-transporting layer (HTL) before deposition of the top Au electrode by sputtering. The complete procedures pertaining to sample preparation are detailed in the experimental section. Figure 1(a) summarizes the overall device architecture for the planar PSC (FTO/ETL/Perovskite/HTL/Au). Figure 1(b) shows a schematic representation of the energy level diagram of the solar cells, with the energy barriers at the interfaces between c-TiO_2_/Perovskite/Spiro-MeOTAD. The perovskite film has a very favorable energy band alignment^8,37^ with the c-TiO_2_ and Spiro-MeOTAD in order to extract electrons and holes respectively. Under solar illumination, perovskite light absorbers generate both charge carriers that traverse the film (thickness ~400 nm), before electrons are eventually extracted by the ETL (50 nm) and holes by the HTL (200 nm).

The thickness chosen for the perovskite layer is compatible with the diffusion lengths of electrons and holes. The latter has been measured to be longer than 1 µm for planar mixed halide perovskite light absorbers^38^. These combined advantages together with their process compatibility and widely accessible deposition methods make these extractors ideal candidates for PSCs. Excluding the final Au electro-deposition by magnetron sputtering, all other device layers are deposited by spin-coating method in ambient conditions.

**Figure 2:**
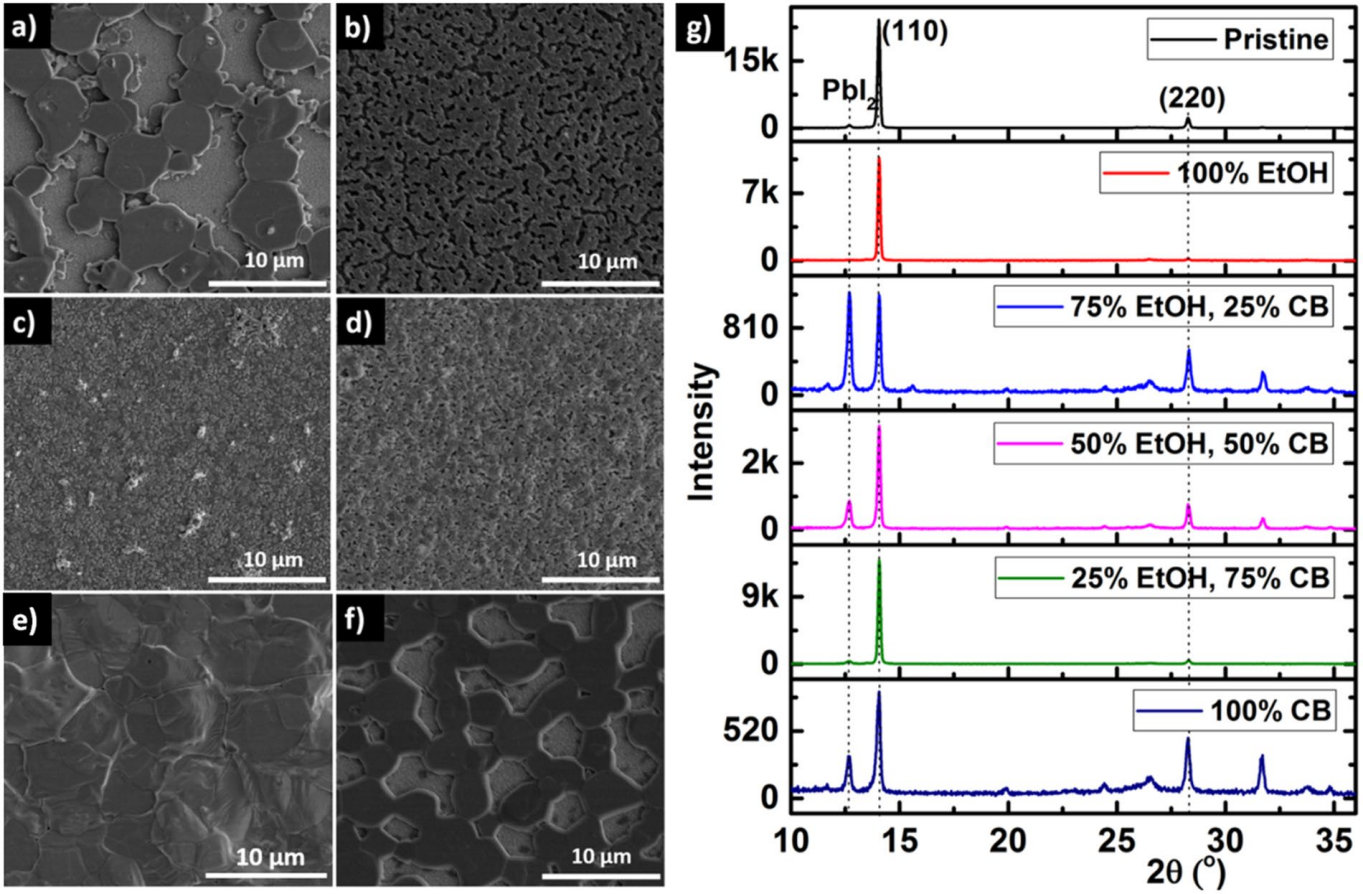
Microstructures of the different halide perovskite films directly on FTO substrate. (**a**) Pristine (as-deposited) film without solvent treatment. (**b**–**f**) Similar films after solvent treatment using (**b**) 100% EtOH, (**c**) 75% EtOH & 25% CB, (**d**) 50% EtOH & 50% CB, (**e**) 25% EtOH & 75% CB and (**f**) 100% CB. (**g**) The XRD spectra corresponding to each perovskite film.

In a one-step deposition of CH_3_NH_3_PbI_3−*x*_Cl_*x*_, the perovskite film shrinks and becomes porous showing significantly large pinholes (~5 µm). Thus a method for crystallization and growth of compact perovskite films is essential to achieve high efficiency solar cells^39^. Our proposal of using a solvent engineering approach originates from achieving a compact film through a solvent controlled crystallization process^39,40^. As depicted in Fig. 1(c), our approach for spin-coating the perovskite layer involves three processing steps: a) 1^st^ spinning for surface coverage, b) 2^nd^ spinning for reducing film thickness and c) 3^rd^ spinning and solvent dripping for crystallization. To achieve larger grains while maintaining a compact perovskite film, chlorobenzene (CB, anti-solvent) and ethanol (EtOH, solvent) were chosen to modulate the film morphology. The SEM images in Fig. 2(a–f), illustrate solvent-engineered perovskite thin films deposited on FTO substrate and treated using different combinations of CB and EtOH. Figure 2(a) shows a pristine un-treated perovskite film with a rough microstructural evolution with large crystal grains (>5 µm) and pinholes. Surprisingly, the SEM analysis first reveals strikingly different morphologies when treated only with EtOH and CB. Indeed, Fig. 2(b) shows a film treated only with EtOH having a much better surface coverage with significantly smaller pinholes size but also significantly reduced crystal grains. In contrast, Fig. 2(f) shows a film treated only with CB having both very large crystal grains (5 µm), but with large pinholes and poor surface coverage. These differences led to the idea of perovskite thin film solvent engineering process using volumetric solvent mixture for micro-structure optimization in order to achieve both larger grains (CB) and good coverage with low pinhole densities (EtOH).

For all treatments, the XRD spectra of the perovskite films are shown in Fig. 2(g). The films exhibit typical peaks of the MAPbI_3_ phase at approximately 14.06° (110) and 28.28° (220). The peak occasionally seen at 12.66° corresponding to the (001) peak of a photo-inactive cubic PbI_2_, indicating a partial decomposition and/or residual unreacted PbI_2_ in the film^21^. However, this residual PbI_2_ completely disappears for samples treated with EtOH alone. For the other samples using EtOH, the intensity of this peak remains relatively weak compared what is observed for the EtOH:CB = 3:1 solvent treatment. This is because a polar solvent such as EtOH dissolves the excess MAI^41^, while the non-polar CB enhances the crystallization process since non-polar CB reduces the solubility of perovskite in the polar DMF that consequently initiates fast nucleation^42^. As EtOH dissolves the remnant MAI, the remaining unreacted PbI_2_ from the first spin-coating step will have a second opportunity to react with MAI during the solvent dripping step.

The XRD pattern from the sample treated with 100% EtOH also reveals a pronounced crystallite orientation along the (110) plane, suggesting highly-oriented crystals with preferential growth direction along this plane^43^. We suggest that such a high degree of order originates from the pre-crystallization step in the presence of a solvent treatment^44^. Yet, CB initiates nucleation and enhances crystallization producing larger crystal grains.

Larger grain sizes in perovskite films would promote the PCE of solar cells since the photogenerated carriers encounter reduced impediments from bulk defects and grain boundaries. Grain boundaries and/or large pinholes sizes or densities are also known to cause poor device performances due to a large number of trap-assisted recombination centers of trapping and lower carrier mobilities^45^. With larger crystal grains the overall performance of the cell will improve since the charge carriers encounter fewer trapping and scattering sources^26,30,46^.

Large pinholes or high densities of pinholes also cause significant recombination events since they can provide a direct contact between the ETL and HTL^47^, limiting the charge extraction efficiency. Based on these findings, we aim to identify an optimal tradeoff by combining EtOH and CB as solvent and anti-solvent respectively to achieve simultaneously large grain sizes and uniformly covered perovskite thin film with minimized pinholes. The film treatment with EtOH:CB at a volumetric ratio of 1:3 results in larger grain sizes (5 µm), fewer pinholes and better surface coverage (Fig. 2(e)) as well as excellent crystalline quality, as seen in Fig. 2(g). In contrast, the sample treated with 3:1 ratio of EtOH:CB shows a compact and nearly pinhole-free surface but with extremely small grain sizes (≤0.5 µm), in addition to poor crystal quality as indicated by the XRD patterns (blue curve in Fig. 2(g) and supporting information S1). As expected, increasing the concentration of CB markedly increases the grain size (Fig. 2(f)), however the size of the pinholes also significantly increases.

The XRD of the perovskite films indicate that all samples match a tetragonal crystal structure with lattice parameters a = b = 8.87 Å and c = 12.65 Å after cell refinement, which is consistent with previous reports^40,48,49^. A more detailed analysis of the phase and stoichiometry for MAPbI_3−x_Cl_x_ has no sign of Cl-based compound or intermediate phase. The conversion of intermediate phases to a fully-crystallized perovskite by annealing was studied systematically using grazing angle X-ray diffraction as shown in Fig. S3. The study was performed at three processing stages: I) as spin-coated, II) after solvent treatment and III) after solvent treatment and thermal annealing. The intermediate phases that are observed in stages I) and II) disappear in stage III (after solvent treatment and subsequent annealing at 125 °C on a hot plate). Here, only peaks from MAPbI_3_ or MAPbI_3−x_Cl_x_ are observed, implying a full transformation of all phases into crystalline perovskite phase. This is consistent with earlier findings^12^, suggesting a strong ion exchange between I^-^ and Cl^-^ and the formation of MACl in the intermediate crystallization step before sublimation during annealing as given in equation (2).

1 $${{\rm{PbI}}}_{2}+{{\rm{PbCl}}}_{2}+4{\rm{MAI}}\to 2{{\rm{MAPbI}}}_{3}+2{\rm{MACl}}\uparrow $$

The absence of other phases such as MAPbCl_3_ in the final perovskite film agrees with other reports, indicating the intercalation of MAI and reconstruction in the intermediate reaction as the main reason for the recrystallization of the intermediate phases^50^. These results are confirmed by micro Raman mapping measurements (Fig. S4) and consistent with previous reports and there is no indication of MAPbCl_3_ in the film. In contrast to our findings, few reports also indicated that the crystallization of MAPbCl_3_ phase^51^. Yet, the presence of Cl in the crystal structure still remains under debate since the detection is beyond the sensitivity limit of many instruments. However, the possibility of a Cl doping in the structure has been proven using DFT calculations and slight volume changes compared to the volume of pure tetragonal MAPbI_3_^52,53^.

To delve further into the quality of the halide perovskite films and their compatibility for device integration, photoluminescence (PL) measurements were carried out at room temperature using 405 nm excitation for perovskite films deposited on ETL buffered FTO. In fact, the PL spectra can give qualitative information on the photo-generated annihilation, recombination and/or transfer to the charge-selective (ETL and HTL) layers.

**Figure 3:**
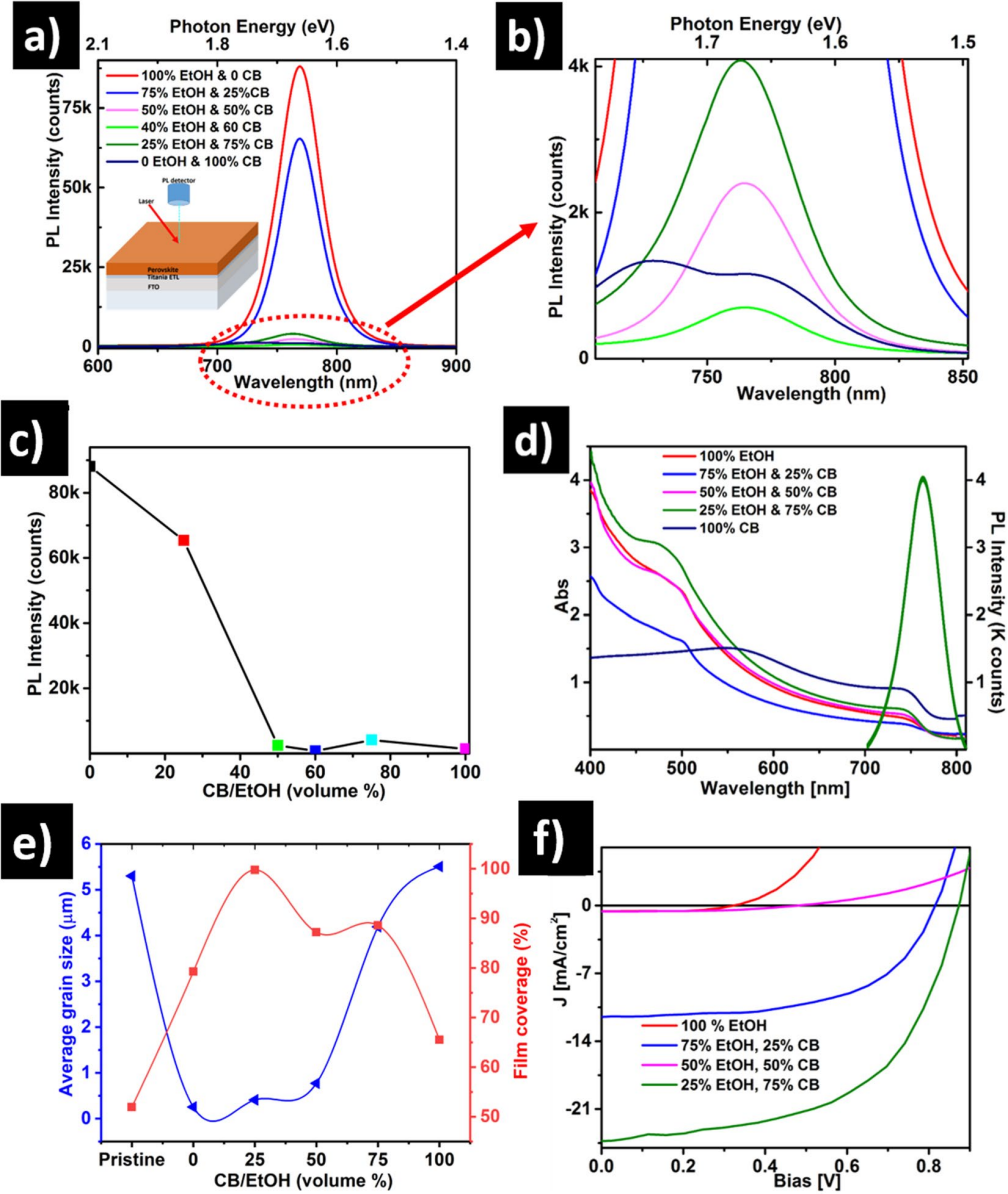
(**a**,**b**) Photoluminescence of perovskite films treated with different mixtures of CB and EtOH. The inset in (**a**) depicts the device architecture used for PL measurements. (**c**) Photoluminescence emission intensity evolution for different solvent treatments. Note: the colored squares in (**c**) match the photoluminescence spectra lines in (**a**) and (**b**). (**d**) UV-Vis absorption spectra after different solvent treatments and photoluminescence of the optimal CB:EtOH = 3:1 treated sample. (**e**) Statistical analysis of the surface coverage calculated from the SEM images of the treated thin films. (**f**) PV performances for PSC devices using various CB:EtOH solvent treatments.

The PL quenching for the films treated with different solvent mixtures is clearly highlighted in Fig. 3(a–c). As expected, all samples display significant emission centered around 763 nm^54,55^. However, the treatment with 100% CB yields a broader PL emission with dominant features at 729 and at 763 nm. The origin of the shoulder may originate from residual PbI_2_, consistent with the XRD results in Fig. 2. The highest PL peak emission is observed in the 100% EtOH treated sample, but the peak intensity decreases by ~25% compared to the sample treated with the 1:3 CB:EtOH solvent ratio. In contrast, the PL emission decreases by ~99% when treated with 3:2 CB:EtOH solvent ratio. This significant drop in PL intensity can be attributed to a decrease in the annihilation of charge carriers at the radiative trap states. Sharp fall in PL intensity is consistent with SEM images as shown in Fig. 2, and a significant change in morphology is observed. A 100% EtOH solvent treatment results in small grain sizes which in turn leads to a high density of grain boundaries and other trap states that cause an increase in emission at the cost of charge carriers. Similarly, PL spectra of other thin films treated with CB:EtOH show a more pronounced reduction of the PL peak. Figure 3(d) compares the optical absorption spectra of the solvent-engineered halide perovskite samples and also shows the PL peak emission wavelength for CB:EtOH = 3:1 on the same curve. The onset of absorption peaks observed are approximately at 763 nm, matching the band edge of the crystalline perovskite and PL peak emission. A significantly higher absorption for longer wavelengths (≥560 nm) is measured for the 100% CB treated sample, because of large grain sizes that induce greater scattering and thereby absorption^56^. However, a significantly stronger optical absorption is measured for the 3:1 CB:EtOH treated sample, which is consistent with XRD results indicating the formation of a superior crystalline film providing a better broadband absorption that can presumably enhance solar cell performance.

Figure 3(e) shows an assessment of the surface coverage and grain size distributions using Image J analysis of the SEM images obtained for different solvent treatments. The pristine film shrinkage yields the highest pinhole area of 180.07 µm^2^ (Fig. S2(a) and Table S1) and poor surface coverage with only 51.9% of the total surface (Fig. 3(f)). The 100% CB treated specimen also yields a relatively poor surface coverage (65.53%). However, a highly-compact perovskite film can be achieved using the CB:EtOH = 1:3 treatment, as confirmed by its 99.9% surface coverage. However, the majority of the grains are below 400 nm in diameter for the CB:EtOH = 1:3, compared to more than 4 µm for the pristine or 100% CB treatments. Interestingly, an optimal co-solvent treatment fulfilling the optimal surface coverage and grain size is achieved by CB:EtOH = 3:1 as shown in Fig. 3(e) where the curves of the average grain size and surface coverage curves cross at the higher values.

**Figure 4:**
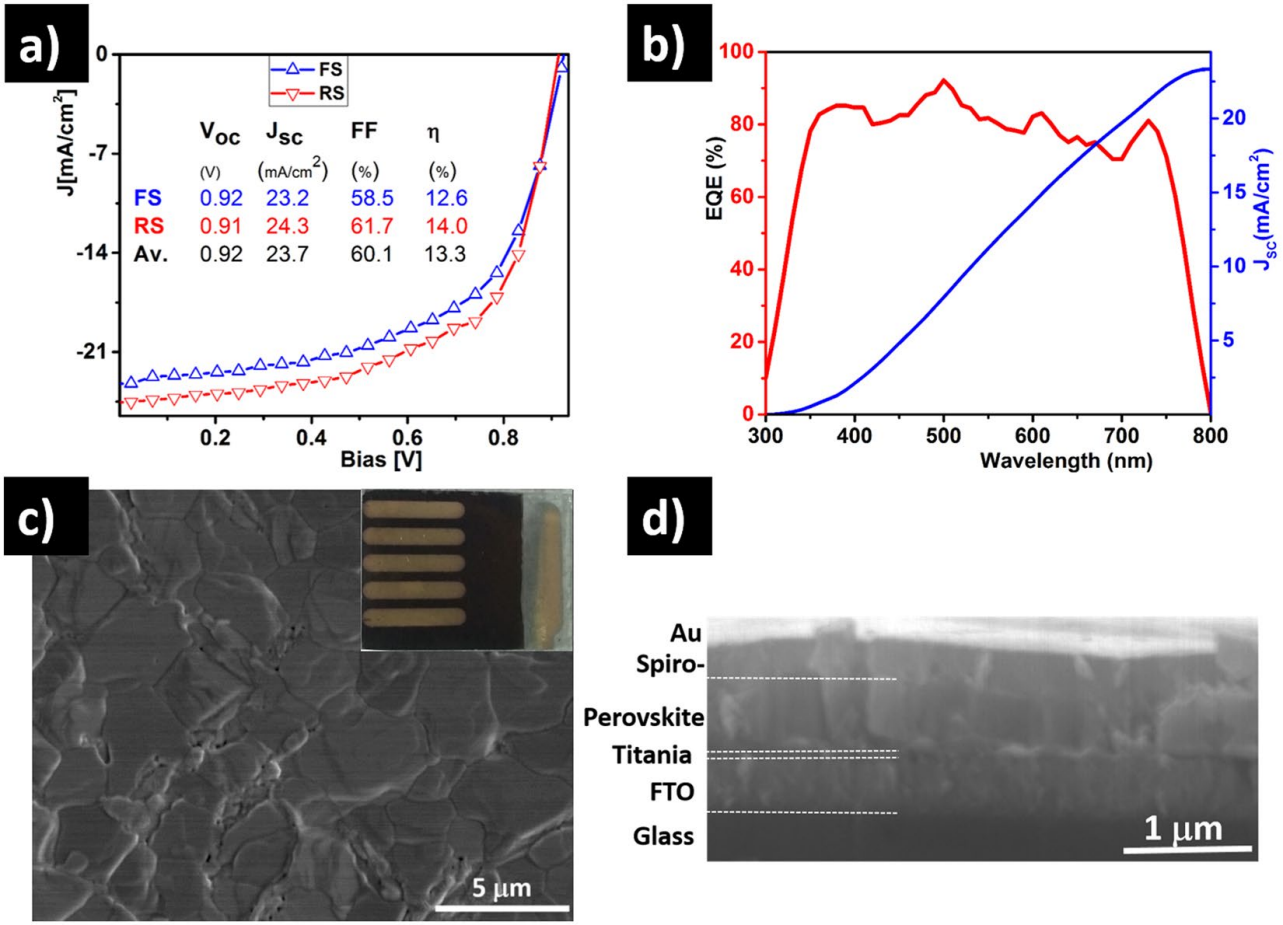
Current density - Voltage (J-V) characteristics of the best-performing cell fabricated using CB:EtOH  =  3:1 solvent treated halide perovskite thin film (C_0.75CB,0.25EtOH_). The J-V curve is measured at 10 mV/s scan speed in both forward-sweep (FS) and reverse-sweep (RS). (**b**) External quantum efficiency (EQE) spectra of the best-performing cell (C_0.75CB,0.25EtOH_). (**c**) Compact perovskite film with larger grains (5 µm) and fewer pinholes for C_0.75CB,0.25EtOH_. The inset shows a picture of the solar cell device. (**d**) Cross-sectional SEM image of the PSC device structure.

Using the optimized CB: EtOH = 3:1 solvent treatment combining large grains with low pinhole densities, planar geometry solar cell devices (C_0.75CB,0.25EtOH_) are fabricated and their PV performances are measured. The current-density versus voltage (J-V) characteristics and the external quantum efficiency (EQE) measurements are shown in Fig. 4(a,b) respectively. The cell shows large open-circuit voltage (*V*_oc_), short-circuit current density (*J*_sc_) and fill-factor (FF) up-to 0.92 V, 23.7 mA/cm^2^ and 61.7%, respectively under 1 sun (100 mW/cm^2^) illumination and voltage scanning rate of 10 mV/s. The best cell exhibits a PCE of 14% in reverse voltage sweep (RS), with only a slight deviation from a forward sweep (low-hysteresis). The EQE measurement confirms the excellent light harvesting quality of the cell, exceeding 80% from 350 to 745 nm even though this measurement was taken 3 weeks after fabrication. The maximum EQE (90%) is even higher than the maximum transmission of bare FTO (85%) (Fig. S6), simply indicating that the reflection is significantly reduced due to the lower step in refraction index from adding the perovskite layer, which still reduces the apparent EQE value. As depicted in Fig. S2(b), some solar cells fabricated using the same processing technique as our best device performed slightly better in terms of hysteresis, but slightly lower in PCE. The *J-V* characteristics of the best cell in the dark and under 1 sun irradiation is measured (Fig. S5(a)). A modest enhancement in PV performance can be observed when the illuminated area is reduced as shown in Fig. S5(b). While we observe an increase in J_sc_ under reduced area, the hysteretic behavior is also more pronounced. This might be related to the difference in the density of pinholes in reduced area conditions. Discrepancies between the forward and reverse sweeps is dependent on various parameters including scan rate, voltage bias and light-doping. In particular the hysteresis behavior in planar PSC has been thoroughly investigated, as it is one of the key parameters for performance comparison^46,57–60^. The trapping and de-trapping process at the interface and grain boundaries, ferroelectric properties, iodine ion movements and other causes within the perovskite materials are claimed to be responsible for the hysteretic behavior and lower extraction efficiencies^61^. Although the cause of the hysteresis remains debatable, it was suggested that iodine ion movements are likely to be one of the main factors^58^.

To confirm the correlation between film morphology and device performance, we can compare solar cells fabricated using different CB:EtOH solvent treatments (Fig. 3(f) and Table S2). To avoid the influence of factors such as humidity and temperature variations, all the samples were fabricated within a few minutes in ambient air conditions. As expected, these measurements confirm that the change in grain morphology (shown in Fig. 3(f)) affects significantly the overall performance of the devices. The lower PCE is recorded when the samples are treated with 100% EtOH, while the highest PCE is achieved when the film is treated with CB:EtOH at 3:1 volumetric ratio. As detailed in Table S2, the improved cell efficiency is mainly prompted by an improvement of open-circuit voltage and short-circuit current density, with FF reaching 60%. As expected from the SEM image in Fig. S1(d), the compact perovskite thin film delivered a larger FF despite the very low short-circuit current density and PCE, which is attributed to increased losses of charge carriers occurring at the numerous grain boundaries^62,63^. The shunt resistance, which is known to reduce the *V*_*oc*_ and FF, seems to significantly affect the performance of all samples. The incomplete coverage of the perovskite film (cf. Fig. 2, 3 and S1) causes the formation of shunt pathways accompanied by an internal charge carrier recombination. These results confirm that the PCE depends strongly on film coverage, pinhole density and crystal grain sizes.

**Figure 5:**
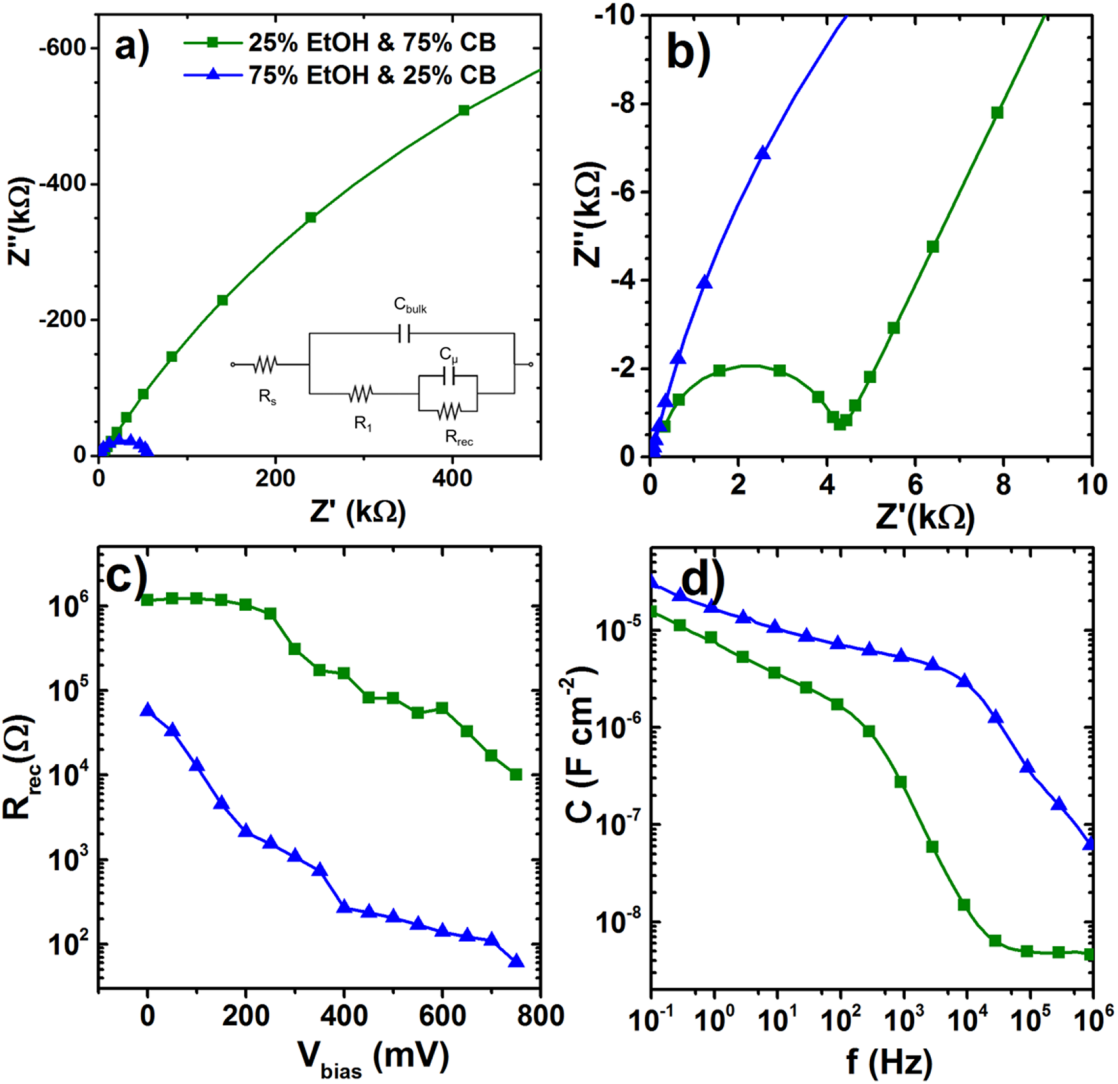
EIS analysis of two PSCs: perovskite thin film treated with mixture of solvents EtOH and CB, one with 75% CB & 25% EtOH (C_0.75CB,0.25EtOH_) and another one with 25% CB &75% EtOH. (**a**) Nyquist plot in dark at 0V. The inset in (**a**) shows the equivalent circuit. (**b**) A zoom-in of the Nyquist plot in (**a**). (**c**) Recombination resistance from EIS measurements in dark. (**d**) The real part of the capacitance as a function of the frequency for the two cells measured in the dark at 0 V bias.

Electrochemical Impedance Spectroscopy (EIS) was also employed on representative samples to probe the electronic properties and the interfacial recombination within the solar cells and their relation to the perovskite film surface morphologies resulting from different CB:EtOH treatments (Fig. 5). For the interpretation of the spectra, we used a circuit model (inset in Fig. 5(a)), to account for the chemical-physical processes taking place at the different interfaces in the PSC. Figure 5(a,b) shows the Nyquist plot of the complex impedance for the two heterojunction solar cells under 0 V biasing in dark, presenting characteristic impedance patterns with two arcs for both cells.

The arc at high frequencies in Fig. 5(b) is generally associated with the geometrical capacitance (*C*_bulk_) in parallel with an equivalent resistance that takes into account the carriers’ conductivity^64,65^.The second arc at medium frequencies is related to the recombination processes inside the solar cell^57,58,66^. As visible in Fig. 5(a), the most remarkable feature is that the best cell fabricated using CB: EtOH = 3:1 treatment (C_0.75CB,0.25EtOH_) leads to the larger arc indicating reduced recombination events. This is consistent with the J-V measurements that show a higher efficiency for this cell compared to the 25% CB & 75% EtOH (CB:EtOH = 1:3) treated cells. Further confirmation arises from the analysis of the recombination resistance (R_rec_) when varying the voltage bias (Fig. 5(c)). At any V_bias_, the value of R_rec_ for the C_0.75CB,0.25EtOH_ cell is at least 50× compared to the 25% CB, 75% EtOH cell. The value of R_rec_ is inversely proportional to the charge recombination rate, so the higher value in the C_0.75CB,0.25EtOH_ cell also indicates a significant suppression of the recombination rate. This substantially improves the injection of electrons in the photoanode, resulting in an increase in *J*_sc_ as shown in Fig. 4(a,b). The EIS results support the interpretation of the PL measurements, indicating that a reduction in grains size significantly increases the probability of recombination at the grain boundaries, thereby reducing the PCE of the solar cells.

Another interesting feature that can be extrapolated from the EIS is the general behavior of the overall capacitance. Figure 5(d) displays the spectrum of the real part of the capacitance in the dark at short-circuit conditions. While the best 75% CB, 25% EtOH-treated perovskite thin film presents a well-defined plateau at high frequencies, this plateau feature is not visible for the 25% CB,75% EtOH. This capacitance can be identified as the bulk capacitance *C*_bulk_ of the perovskite layer, from which we can calculate the dielectric constant ε_r_ of the perovskite thin film. Considering a 400 nm thick film and a *C*_bulk_ = 50 nF/cm^2^, we obtain ε_r_ = 22.5, which is consistent with the values previously reported for similar systems in the literature^65,67^. At medium frequencies, we observe a shoulder that can be identified as the chemical capacitance, in addition to a static dielectric capacitance at low frequencies identifiable for both cells. The giant capacitance value observed at low frequencies is consistent with previous reports and reflects the ion migration to the electrodes and charge compensation by the external electrodes^65,68^. The main difference between the two cells is the absence of the plateau at high frequencies for the 25% CB, 75% EtOH cell that shows a broadening of the chemical capacitance up to 1 MHz. This effect has been previously reported for similar devices and is related to the high roughness at the interfaces between thin-film multilayer contacts^65,69^. This confirms again the difference in the thin film morphology indicating a higher roughness at the planar heterojunctions as a result of smaller grain sizes and higher grain boundaries for the 25% CB and 75% EtOH treated cell. Besides SEM and PL, EIS gives additional supporting evidence of grain size and grain boundaries in the film being the main factors influencing the PCE of such perovskite-based solar cells.

## Methods

### Etching FTO and deposition of blocking layer

A Fluorine-doped tin oxide-coated glass substrate (Tec 8, Osilla Limited) was partly etched using Zn paste and 37% concentrated HCl (Sigma-Aldrich Canada Co.) diluted in deionized water (HCl to deionized water volume ratio 1:3). After etching FTO, we followed a three step ultrasonic cleaning procedure: (i) 20 min ultrasonication in deionized water (5% detergent) mix, followed by rinsing in deionized water, (ii) 5 min ultrasonic bath in acetone and dried in air, (iii) 5 min ultrasonic bath in Isopropanol, rinsing in deionized water and drying with pressurized nitrogen gas blowing.

UV-Ozone (UVO, Ossila Ltd) was used to clean the surface for 20 minutes before spin-coating the blocking layer so that possible contaminants are removed completely besides improving surface hydrophilicity. A TiO_2_ blocking layer composed of 20-nm-sized particles (Ti-Nanoxide BL/SC TiO_2_ BL, Solaronix SA) was then deposited by spin-coating at 5000 r.p.m. for 30 s followed by a crystallization heat treatment at 550 °C for 45 minutes. The compact TiO_2_ is deposited on FTO from a commercially available Titania solution which is spin-coated and annealed using the recommended method from the company.

### Preparation of Perovskite Precursor Solution

Unless stated otherwise, all materials and solvents used for the preparation of the halide perovskite precursor were purchased from Sigma-Aldrich. The precursor is prepared by using commercially available PbI_2_, PbCl_2_ and MAI (Solaronix SA). The solution is prepared in DMF, (99.8%) solvent in a molar concentration of 0.5M PbI_2_, 0.5M PbCl_2_, 2M MAI. A bright yellow and transparent perovskite solution is obtained by the addition of 2% (by volume) of a 36.5–38% concentrated HCl into the solution and stirring 20 minutes.

### Device Fabrication

A planar heterojunction device is fabricated in the following order multilayer stacking order: FTO/compact TiO_2_/perovskite/Spiro-MeOTAD/Au. The patterned FTO glass substrates coated with compact TiO_2_ were first treated with UV Ozone for 5 minutes to remove possible impurities, followed by spin-coating of 120 µl perovskite solution at three step spin-coating speed: 1000 rpm for 10 s, 2000 rpm for 20 s and 6000 rpm for 15 s. The mixed solvents described above were prepared for various concentration ratios and 300 µl are applied in each case immediately at the beginning of 6000 rpm spinning. The spin-coated samples are then annealed at 125 °C for 15 minutes for complete crystallization. We then use the highly efficient hole extracting organic material spiro-MeOTAD which is prepared by following an existing procedure^70^. A volume of 40 µl of spiro-MeOTAD solution is spin-coated at 4000 rpm for 30 s, after the samples are brought to room temperature. The solution is prepared in ambient conditions by dissolving 144.6 mg of sublimed spiro-MeOTAD (99.5% purity, FrontMaterials Co., Ltd) dissolved in 2 ml chlorobenzene and then added 56.6 μl of 4-tert-butylpyridine, 35 μl lithium bis (trifluoromethanesulfonyl)imide (LiTFSI) solution (520 mg LI-TSFI in 1 ml acetonitrile, 99.8%) and 58 μl of tris(2-(1H-pyrazol-1-yl)-4-tertbutyl pyridine) cobalt(III) bis(trifluoromethylsulphonyl) imide (FK209, Dynamo) solution (300 mg FK209 in 1 ml acetonitrile). The humidity during all synthesis and fabrication processes was around 40% and the temperature ranged between 20 and 25 °C. Finally, after overnight storage of the sample in atmospheric conditions, 80 nm gold is sputtered deposited at a rate of 0.2 nm/s (Cressington 208 HR Sputter coater, Ted Pella Inc).

### Device Characterization

The microstructural evolution and morphology of the perovskite thin films are characterized using scanning electron microscope (SEM, Joel JSM-6300F: Japan). The crystallinity and phase of the perovskite films are determined by X-ray diffraction (XRD, D8 Advance: Bruker). The *J–V* characteristic curves of the PSCs are measured using a high precision sourcing and measuring instrument (Keysight B2901A precision source and measuring unit) under AM 1.5 illumination at 100 mW cm^−2^ (cell to simulator distance of ~16 cm) of a solar simulator (SLB 300A, Ocean Optics Inc.) under irradiance of the active cell area of 5.75 mm^2^. The UV-Vis absorption spectra of the perovskite films are recorded on a UV-Vis spectrophotometer (Lambda 20: Perkin Elmer).

The EQE of the devices are measured at room temperature in ambient atmosphere using a monochromator equipped with a xenon arc lamp. The monochromatic light is chopped at a frequency of 30 Hz and focused onto the sample. The active area is defined using a circular aperture with a diameter of 0.5 mm. The photocurrent at each wavelength *I*(λ) is measured with a lock-in amplifier (Ametek 1256) at 0 V. The light power at each wavelength *P*(λ) is measured, through the same aperture, with a calibrated photodiode (Newport 918D) placed at the same position of the device. Then, the EQE(%) value at each wavelength is determined using the following equation:

2 $${\rm{EQE}}( \% )=100hcI\,({\rm{\lambda }})/q{\rm{\lambda }}P({\rm{\lambda }})$$

where *c* is the speed of light, *h* is the Planck constant and *q* is the electronic charge.

EIS was conducted in dark using a SOLARTRON 1260 A Impedance/Gain-Phase Analyzer. The applied AC signal is 10 mV in amplitude, in the frequency range between 0.1 Hz and 1 MHz. The applied bias during measurements is between 0 V and 800 mV. All the samples are measured inside a Faraday cage. The obtained spectra are fitted with Z-View software (v3.0, Scribner Associate, Inc.) by applying an appropriate equivalent circuit.

## Conclusions and Perspectives

We presented a new mixed solvent treatment approach at ambient condition for synthesizing highly oriented perovskite grain crystallites with statistically controlled sizes and pinhole densities that significantly affect the performance of the perovskite-based solar cells. Crystal grains larger than 5 µm with minimal grain boundaries and lowest pinhole densities are achieved using an optimized solvent treatment using a mixed 75% CB and 25% EtOH solvent solutions. The devices are fabricated by sandwiching a halide perovskite thin film between charge-selective layers entirely in ambient air without mesoporous scaffold. The solar cell performances are analyzed and characterized using SEM micrographs, photoluminescence quenching and EIS measurements. In a simple planar geometry solar cells, we find that the density and size of the pinholes are the dominant factors, which affect the performance of the solar cells. Our work defines a liquid solvent processing approach that can be further developed for large area thin film deposition and mass production of high quality perovskite films for PV or other optoelectronic applications. This methodology could be further developed into a vapor phase solvent technique, potentially allowing to obtain a more homogeneous and smooth film through a vapor solvent crystallization process. Our method is promising for cost-effective processing of perovskite-based solar cells.

## Electronic supplementary material

Supplementary information
