# Supplementary material for: Solvent-Antisolvent Ambient Processed Large Grain Size Perovskite Thin Films for High-Performance Solar Cells
Source: Sci Rep. 2018 Aug 27;8:12885. doi: 10.1038/s41598-018-31184-0 (PMC6110839; doi:10.1038/s41598-018-31184-0)
Supplement: Supplementary file 1 — Supplementary information [file 41598_2018_31184_MOESM1_ESM.pdf]

Supplementary Information:

Solvent-Antisolvent Ambient Processed Large Grain Size Perovskite Thin Films for High-Performance Solar Cells

Dawit Gedamu, [*ab] *Ivy M. Asuo,*[ab] *Daniele Benetti,* [b] *Matteo Basti,*[b] *Ibrahima Ka,*[a] *Sylvain G. Cloutier,*[a] *Federico Rosei,* [*ac,] *Riad Nechache,*[*a]

[a] École de technologie supérieure (ÉTS), Department of Electrical Engineering, 1100 rue Notre-Dame Ouest Montréal (QC) H3C 1K3, Canada

[b]INRS-EMT Centre for Energy, Materials and Telecommunication, 1650 Boul. Lionel Boulet, Varennes (QC), J3X 1S2, Canada

[c]Institute of Fundamental and Frontier Science, University of Electronic Science and Technology of China, Chengdu 610054, PR China

* Corresponding authors

E-mail: dawit-minale.gedamu.1@etsmtl.net, rosei@emt.inrs.ca, Riad.Nechache@etsmtl.ca

Keywords: hybrid perovskites, solar cells, highly oriented grains, grain size control

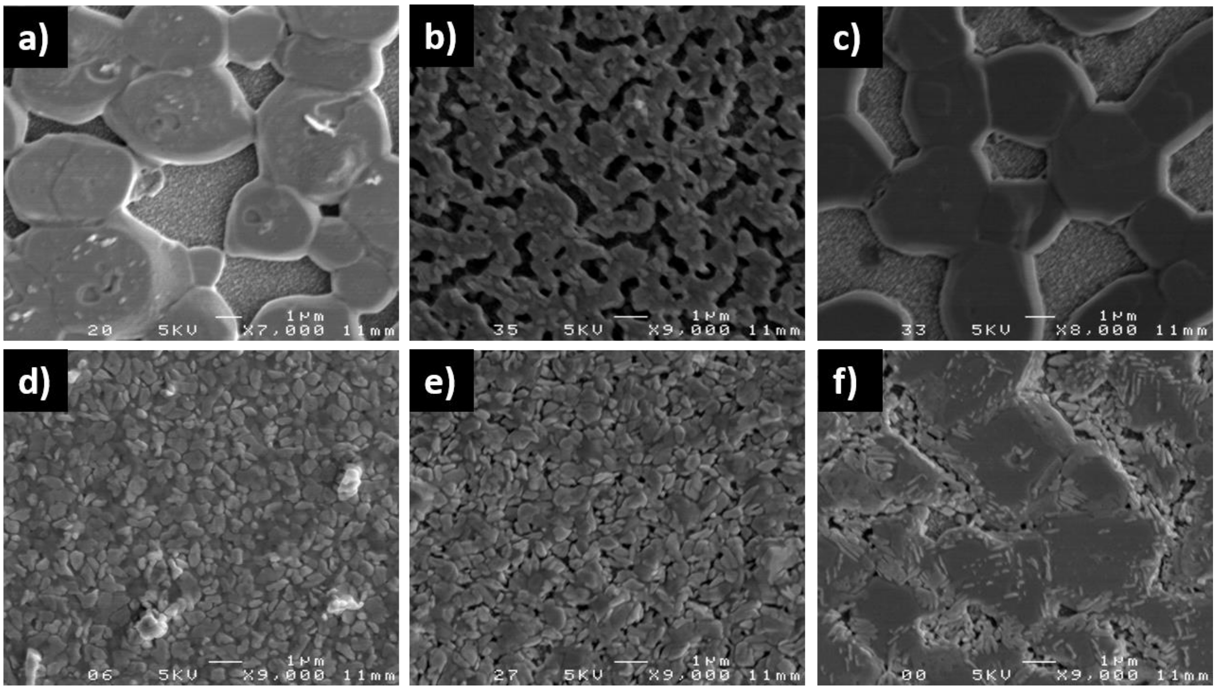

Figure S1: High magnification SEM images for perovskite layer based on 0.5 M PbI_2, 0.5M $PbCl_2$ and 2 M MAI deposited directly on FTO substrate and treated at a pure and a mixed state of solvents in a volume ratio EtOH and CB **a)** Pristine **b)** 100% EtOH **c)** 100% CB **d)** 25% CB to 75% EtOH, **e)** 50% CB to 50% EtOH **f)** 75% CB to 25% EtOH.

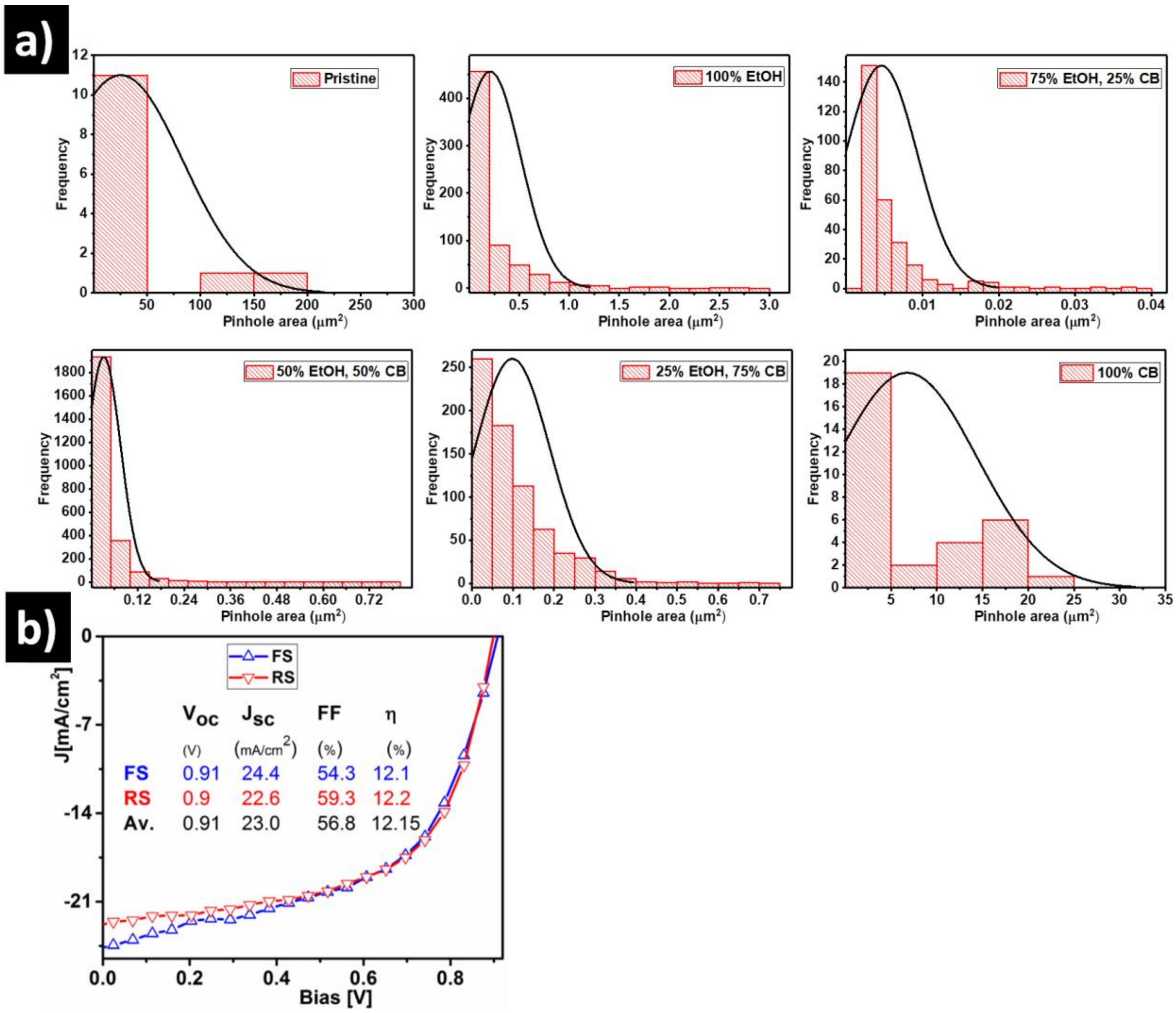

Figure S2: (a) Statistical analysis of pinhole size and distribution for different co-solvent treatments and (b) J-V characteristic of perovskite $C_{0.75CB,0.25EtOH}$ solar cell with the lowest hysteresis

Table S1: Summary of the pinhole surface coverage on perovskite thin films synthesized at different co-solvent treatment corresponding to Figure S2(a)

CB/EtOH (volume ratio %)	Total film area (μm^2)	Total Pinhole area (μm^2)	Maximum size (μm^2)	Minimum Size (μm^2)	Average Size (μm^2)	% Pinhole area	SD
Pristine	681.88	327.55	180.07	0.002	25.21	48.06	58.36
0	659.45	136.7	2.74	0.002	0.20	20.73	0.31
25	651.07	1.35	0.036	0.002	0.01	0.24	0.01
50	601.19	77.00	0.77	0.002	0.03	12.81	0.04
75	611.62	69.84	0.65	0.001	0.10	11.42	0.09
100	626.19	215.85	22.47	0.002	6.75	34.47	7.66

Table S2. PV performance in RS for different solvent mixture treatments of the perovskite film extracted from Figure S2(b)

CB/EtOH (volume ratio %)	J_{sc} (mA/cm^2)	V_{oc} (V)	FF (%)	η
0	0.32	0.435	57.06	0.1
25	9.96	0.82	59.83	4.9
50	0.62	0.57	53.31	0.2
75	24.1	0.88	56.30	11.9

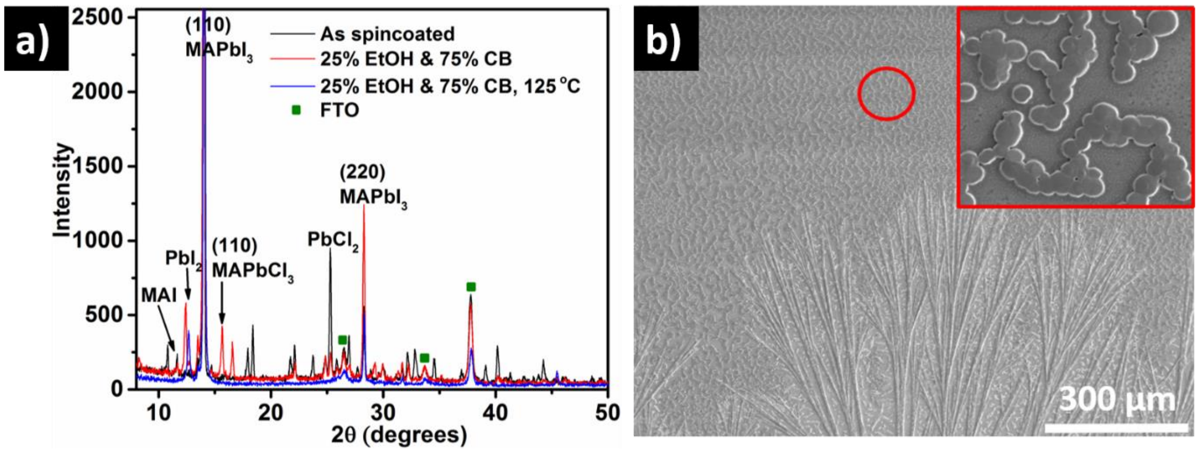

Figure S3: Halide perovskite layer based on 0.5 M PbI_2, 0.5M $PbCl_2$ and 2 M MAI deposited on FTO substrate, a) Major peaks at various solvent treatment conditions, unnamed peaks show different types of intermediate phases b) SEM image as spin-coated perovskite thin film after ambient condition drying: the inset shows a magnified image of a region within the red circle.

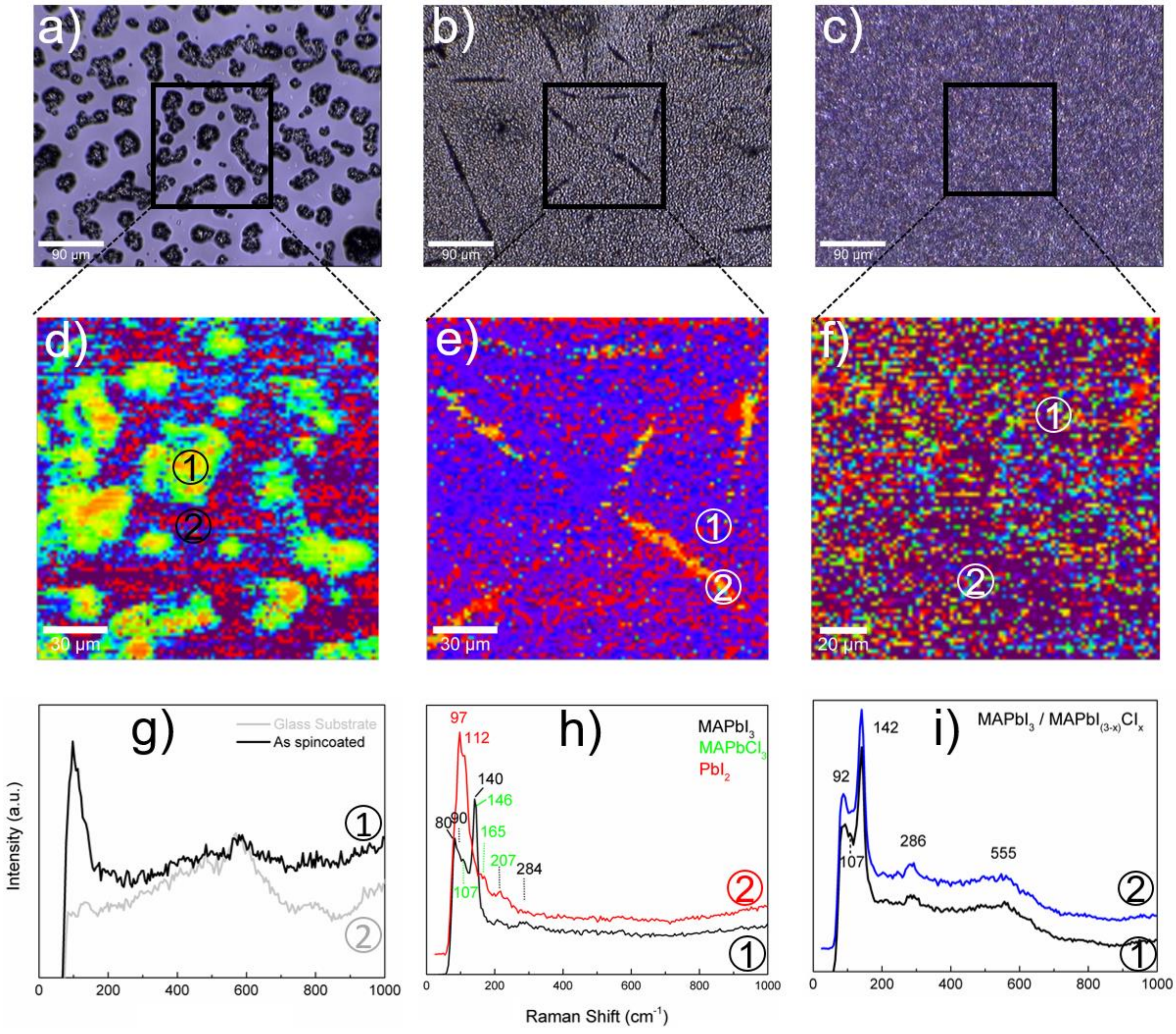

Figure S4: Optical microscopy images and corresponding micro- Raman mapping and spectra for perovskite layers based on 0.5 M PbI_2, 0.5M $PbCl_2$ and 2 M MAI deposited on FTO substrate and obtained at different synthesis steps: (a,d,g) as spin-coated, (b,e,h) after solvent 75% CB, 25% EtOH treatment before annealing and (c, f, i) after solvent treatment and thermal annealing at 125ºC for 15 min.

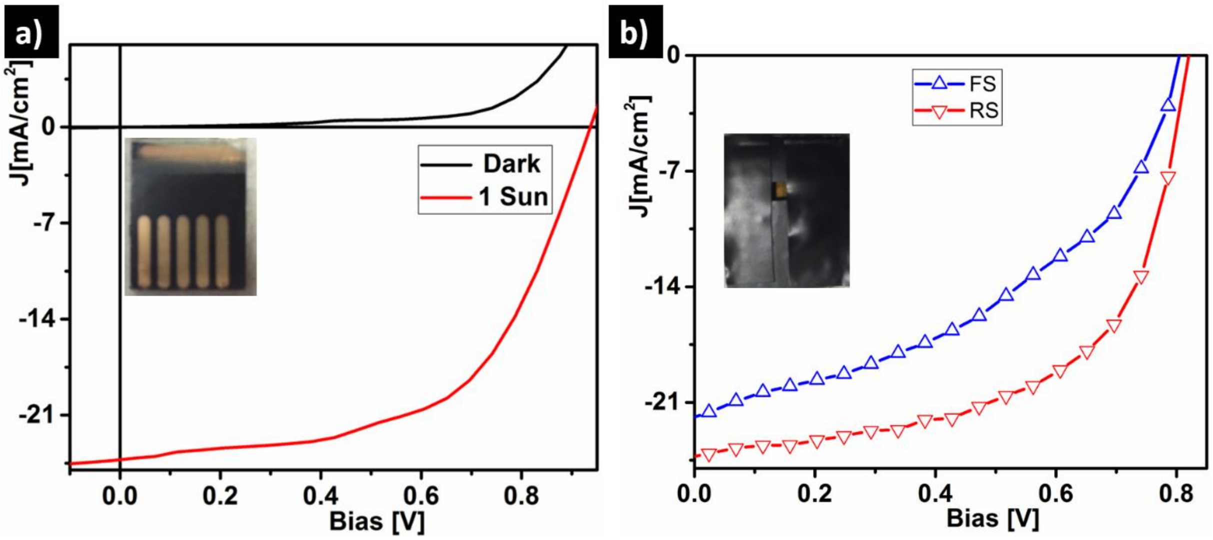

Figure S5: *J-V* characteristic of the best-performing solar cell, a) comparison in dark and under solar illumination for the actual cell shown in inset image (5.75 mm^2), b) under solar illumination of reduced area (2.074 mm^2) by covering the rest of the solar cell area (as shown by the camera image in the inset).

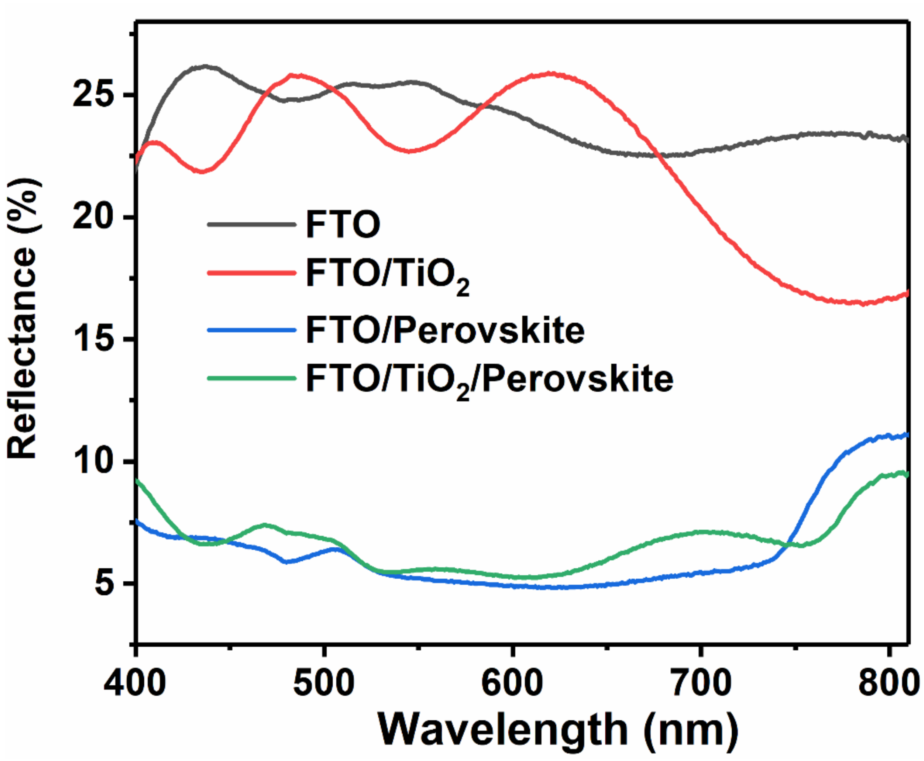

Figure S6: Reflectance measurements for bare FTO and after multi-layer depositions.

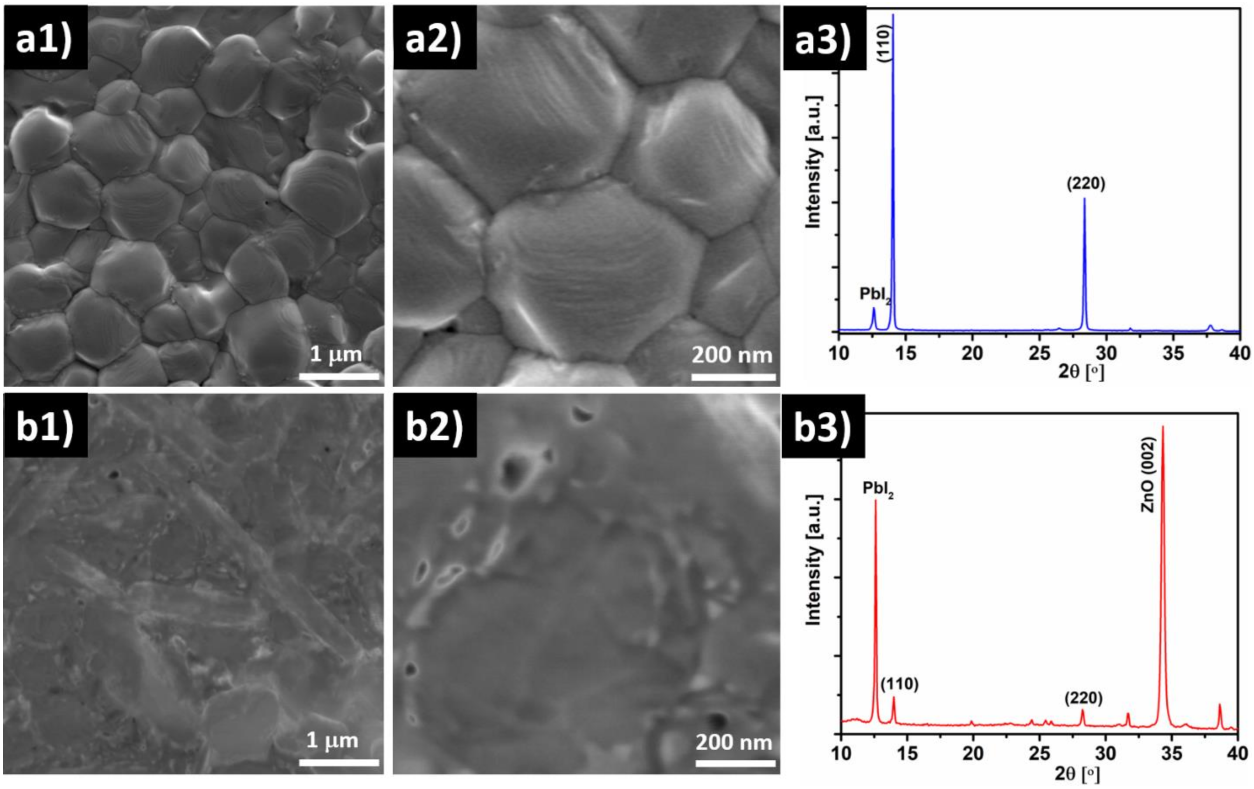

Figure S7: Effect interfaces on the microstructural evolution and crystallization of halide perovskite: where a1) and a2) are SEM images and a3 shows the XRD pattern for NiOx interface. The figures b1) and b2) show SEM images perovskite layer on ZnO interface and b3) shows the corresponding XRD pattern. The perovskite films in both cases are processed in the same condition by annealing at 125 °C for 15 minutes after CB:EtOH = 3:1 treatment.

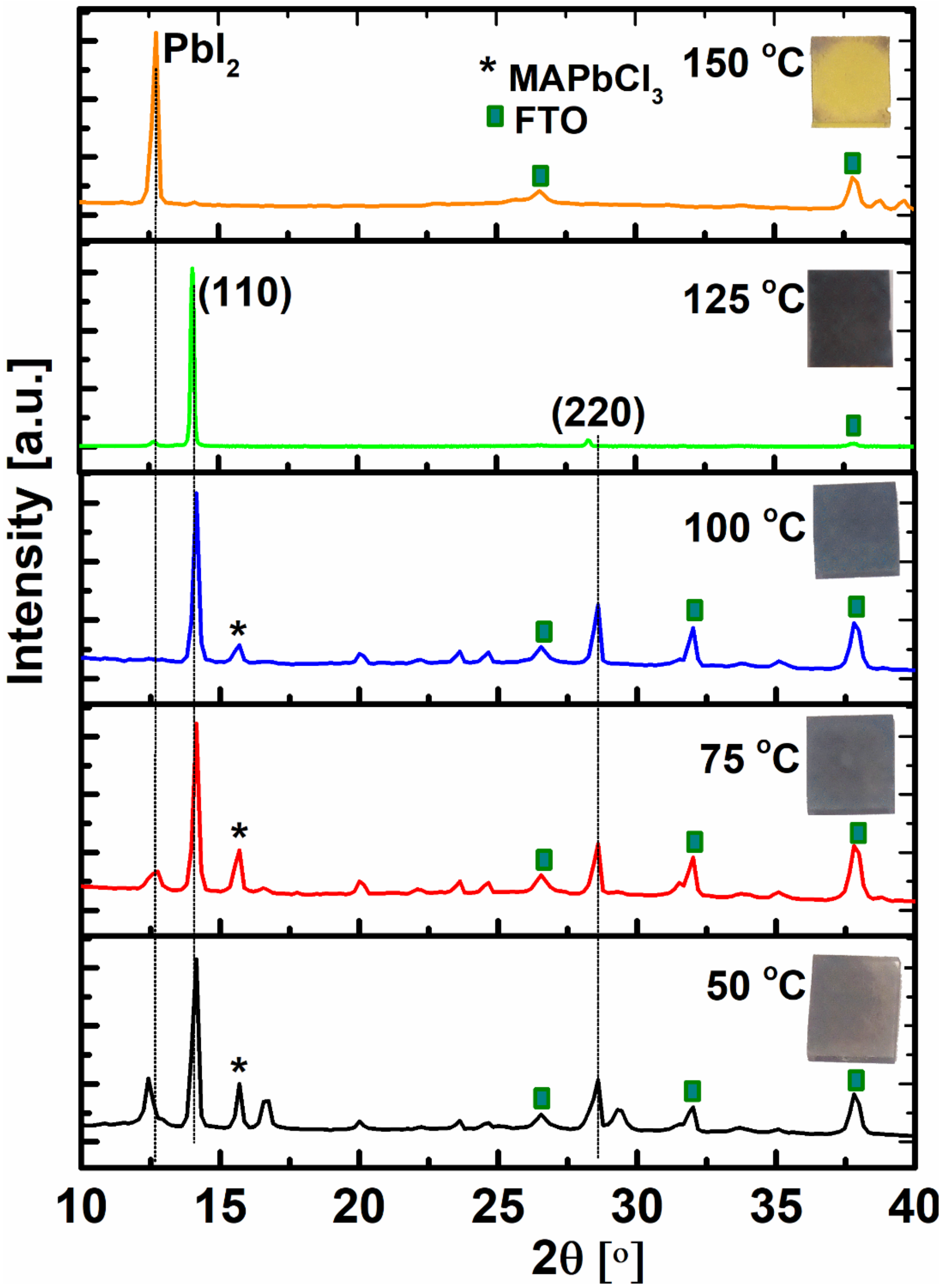

Figure S8: Effect of temperature on the crystallization of halide perovskites film annealed after CB:EtOH = 3:1 treatment.
